# Synergistic interactions of ruthenium-based carbon monoxide-releasing molecules and antibiotics in their effects on Escherichia coli

**DOI:** 10.1099/mic.0.001669

**Published:** 2026-02-05

**Authors:** Salar Ali, Lauren K. Wareham, Robert K. Poole, Samantha McLean

**Affiliations:** 1 Department of Molecular Biology and Biotechnology, The University of Sheffield, Sheffield, S10 2TN, UK; 2 Department of Ophthalmology and Visual Sciences, Vanderbilt University Medical Center, Nashville, TN 37232, USA; 3 School of Science and Technology, Nottingham Trent University, Nottingham, NG11 8NS, UK

**Keywords:** antibiotics, carbon monoxide, carbon monoxide-releasing molecules (CORMs), drug interactions, *Escherichia coli*

## Abstract

The emergence of antibiotic-resistant pathogenic bacteria poses a major and growing public health risk. Because antibiotics act on specific molecular targets, bacteria may evolve resistance mechanisms that alter or bypass these targets. This work investigated the antimicrobial effects of carbon monoxide-releasing molecules (CORMs) and their potential for co-administration with a variety of commonly used antibiotics against *Escherichia coli*. CORMs, commonly transition metal carbonyls, release carbon monoxide under certain conditions. Interestingly, CORMs have been shown to exert antimicrobial activity against bacteria both *in vitro* and *in vivo*. However, to effectively treat patients with antibiotic-resistant infections, combination therapies involving two or more antimicrobial agents may be a useful approach. Herein, we report the antimicrobial activity of CORM-2 and CORM-3 against *E. coli* and, importantly, that sub-inhibitory concentrations of either compound in combination with antibiotics showed a significant increase in efficacy of the conventional antibiotics as assessed by inhibition of bacterial growth and reduced viability. Furthermore, administration of sub-inhibitory concentrations of CORMs increased the antimicrobial activity of multiple antibiotics with a range of modes of action when measured by E-tests and microdilution broth assays. The minimal bactericidal concentrations were reduced 2- to 8-fold and 10- to 63-fold for CORM-2 and CORM-3, respectively. Drug interactions between CORMs and antibiotics were also assessed using checkerboard microdilution methods, providing evidence that CORM activity is synergistic with a wide range of conventional antibiotics tested with fractional inhibitory concentration indices between 0.31 and 0.45. These findings demonstrate the antibacterial activity of CORMs and their synergy with a range of commonly used antibiotics, opening potential avenues for CORMs to be used as adjuvants to traditional antibiotic treatments.

**Impact Statement**

The global rise of antibiotic-resistant bacteria presents a critical challenge to public health, requiring innovative strategies to enhance antimicrobial efficacy. This study explores the potential of carbon monoxide-releasing molecules (CORMs), specifically CORM-2 and CORM-3, as adjuvants to conventional antibiotics against *Escherichia coli*. While CORMs are known for their antimicrobial properties, this work uniquely demonstrates their synergistic effects when used at sub-inhibitory concentrations alongside a range of antibiotics. The findings reveal significant reductions in bacterial viability and minimal bactericidal concentrations, with synergy confirmed through checkerboard assays. This research adds to the growing literature on non-traditional antimicrobial agents by highlighting a novel, broad-utility approach to potentiate existing antibiotics. The implications are far-reaching: CORMs could be integrated into combination therapies to combat resistant infections more effectively, potentially revitalizing the clinical utility of antibiotics compromised by resistance. This represents a meaningful step forward in antimicrobial strategy, offering a promising adjunctive treatment avenue that could be translated into clinical settings.

## Introduction

Antimicrobial resistance is a major global health challenge. In May 2014, the World Health Organization warned that antimicrobial resistance had reached alarming levels worldwide, with increasing prevalence of multidrug-resistant organisms and treatment failures reported across major regions [1]. More recently, a landmark study estimated that antimicrobial resistance was directly responsible for 1.27 million deaths globally in 2019, surpassing the mortality burden of HIV/AIDS and malaria [2]. Urinary tract infections are predominantly caused by uropathogenic *Escherichia coli* [3,4], which can form intracellular bacterial communities [5]. The ability of uropathogenic *E. coli* to produce biofilms further contributes to its persistence within the urinary tract [6]. A key mechanism of resistance among *Enterobacteriaceae* is the production of extended-spectrum *β*-lactamases, which confer resistance against cephalosporins [7]. In addition, *Enterobacteriaceae* have been reported to exhibit resistance to multiple classes of antibiotics, leading to the emergence of multidrug-resistant strains [8].

Antibiotics are the standard treatment for most bacterial infections, including urinary tract infections [9]. However, resistance to many commonly used antibiotics has been identified in uropathogenic *E. coli* strains. To address this growing challenge, it is essential to explore alternative compounds for antimicrobial activity against multidrug-resistant pathogens. Non-antibiotic antimicrobial agents may offer a promising strategy by enhancing the effectiveness of existing antibiotics, potentially restoring their efficacy against resistant bacterial strains.

Carbon monoxide-releasing molecules (CORMs) have demonstrated antimicrobial activity against a range of bacterial species, including *E. coli* and *Salmonella enterica*, as well as against several ESKAPE pathogens, including *Pseudomonas aeruginosa*, *Acinetobacter baumannii* and *Staphylococcus aureus*, with some reports also noting effects on *Enterococcus* species [10,13]. Among these, CORM-2 and CORM-3 are two of the most widely studied compounds. CORM-2 is a ruthenium-based, water-insoluble molecule ([Ru(CO)₃Cl₂]₂) that releases carbon monoxide upon activation, though its solubility in organic solvents such as DMSO limits its pharmacological applications [14]. Despite this, it has shown biological activity, including vasodilatory effects and selective toxicity towards bacterial cells over mammalian cells [14,15]. CORM-3, in contrast, is a water-soluble analogue ([Ru(CO)₃Cl(glycinate)]) that releases CO rapidly at physiological pH [16]. It has demonstrated antimicrobial effects at low micromolar concentrations, including activity against multidrug-resistant *P. aeruginosa*, with minimal toxicity to eukaryotic cells [17].

Given the growing interest in combination therapies to overcome antibiotic resistance, this study investigates the antimicrobial effects of CORM-2 and CORM-3, both alone and in combination with antibiotics of varying mechanisms. We assessed their impact on the growth and viability of *E. coli*, with the aim of evaluating their potential as antibiotic adjuvants. To establish a clear baseline for evaluating CORM–antibiotic interactions, we employed the well-characterized strain *E. coli* MG1655. This model provides a controlled and reproducible system for initial mechanistic insights, forming a foundation for future studies involving clinical and multidrug-resistant isolates.

## Methods

### Reagents

**Fig. 1.**
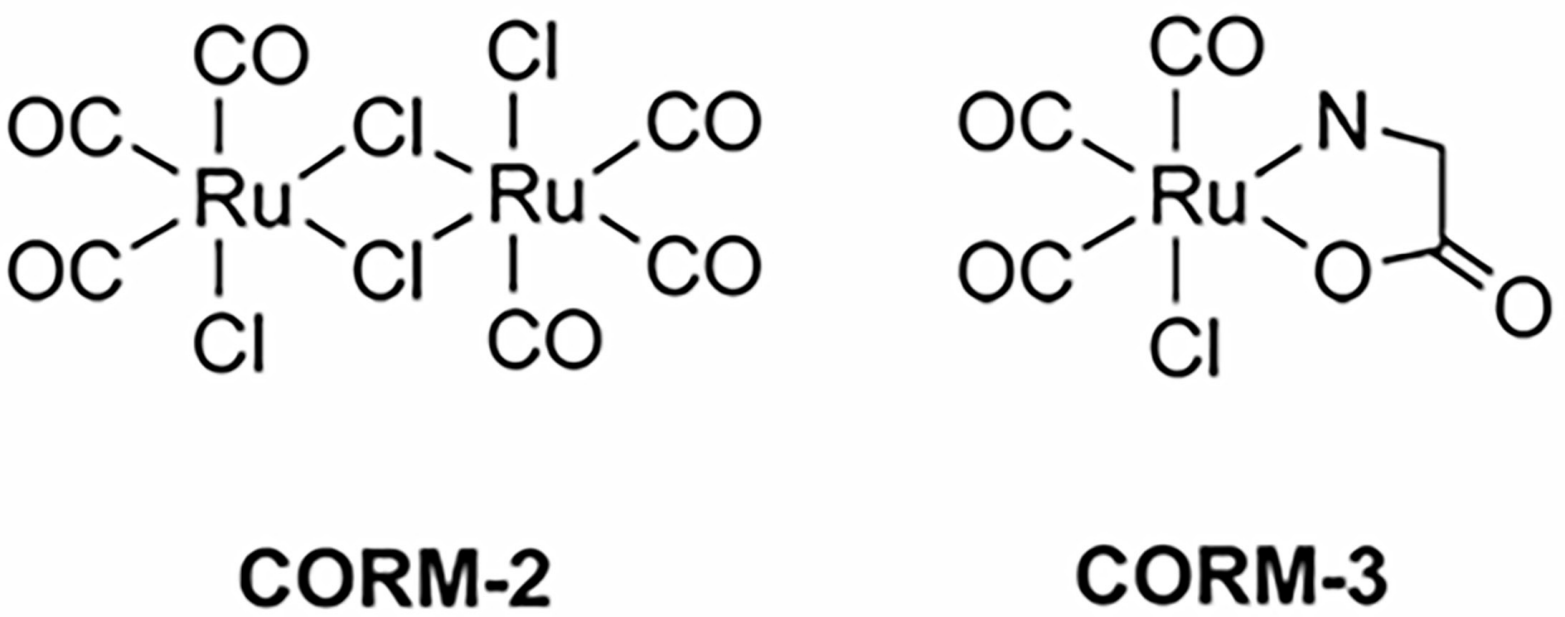
Chemical structures of ruthenium-based CORMs.

Tricarbonyldichlororuthenium(II) dimer (CORM-2) was purchased from Merck, UK, and tricarbonylchloro(glyinato)ruthenium(II) (CORM-3) was kindly donated by Professor Brian Mann (Department of Chemistry, The University of Sheffield). Stocks (10 mM) were freshly prepared by dissolution in DMSO and water, respectively, and stored on ice for use. Stocks were used fresh or on the following day after overnight storage at 4 °C. Their chemical structures are shown in Fig. 1. Doxycycline, minocycline, gentamicin, spectinomycin and cefotaxime were dissolved in water; trimethoprim in DMSO; and chloramphenicol in ethanol prior to use. All reagents were purchased from Merck, UK, unless otherwise stated.

### Bacterial strain, media and growth conditions

*E. coli* strain MG1655 was used throughout [15] and was revived from cryopreserved stocks on Luria–Bertani (LB) agar. Starter cultures were grown overnight at 37 °C in LB broth. Defined minimal medium with 5% glycerol as the sole carbon source was used in all experiments [18]. *E. coli* was incubated at 37 °C in aerobic conditions using a shaking incubator.

### Preparation of control compounds (‘inactive’ CORMs, iCORMs)

The non-CO-containing control for CORM-2, named RuCl_2_(DMSO) or iCORM-2, was provided by Dr Tony Johnson (Department of Chemistry, The University of Sheffield). A solution of this compound (100 mM) was prepared fresh on the day of each experiment in distilled water. For DMSO, a volume equivalent to the 10 µM CORM-2 solution added was used.

The inactive form of CORM-3 (iCORM-3) was made by incubating a fresh CORM-3 dissolved in PBS at room temperature for up to 48 h with periodic N_2_ gas bubbling, after which time <5% CO release was detected.

### Growth and viability assays

*E. coli* starter cultures (10 ml) were centrifuged at 5,000 r.p.m. for 10 min, resuspended in 30 ml defined minimal medium in conical flasks and incubated with shaking at 200 r.p.m. at 37 °C until a turbidity of OD_600_ 0.3–0.4 was reached. Then, varying concentrations of CORMs alone or in combination with antibiotics were added to the cultures. Turbidity was measured every 2 h up to 8 h and at 12 and 24 h, with viability measured at 24 h. For descriptive growth rate comparisons, μmax was calculated as the maximum slope of ln(OD₆₀₀) versus time for each condition.

### BioMérieux E-test MIC assays in the presence or absence of CORMs

E-tests were performed in accordance with the manufacturer’s instructions (bioMérieux, France) with adjustments made to incorporate CORM and control exposure. Briefly, starter cultures of *E. coli* were prepared and adjusted to 0.5 McFarland standard and then centrifuged for 10 min at 5,000 r.p.m. and resuspended in 5 ml defined minimal medium. Diluted culture (50 µl) was added to 4 ml super-soft defined minimal medium agar (0.5% agar), and then immediately CORMs, iCORMs or DMSO were added, mixed and poured onto the surface of defined minimal medium agar plates. The plates were dried for 15 min prior to application of the E-test strips (bioMérieux, France) and were subsequently incubated at 37 °C. MIC values were measured after 24 h. The E-test MIC was defined as the antibiotic concentration at which the border of the elliptical zone of complete inhibition intersected the scale on antibacterial test strip, and the MIC breakpoints for defining interpretive categories published by EUCAST [18] were used for interpreting E-test MIC values.

### Determination of MIC and bactericidal concentration for CORMs and antibiotics using microdilution broth assays

The interactions of CORMs with antibiotics were determined using a broth microdilution checkerboard assay [19]. Briefly, a starter culture of *E. coli* was centrifuged at 5,000 r.p.m. for 10 min and then re-suspended in defined minimal medium to an OD_600_ ~0.05. Aliquots of diluted culture (1.2 ml) were distributed in 24-well plates. For each antibiotic, the following range of concentrations was used with increasing twofold concentrations: doxycycline (0.5–200 µg ml^−1^), minocycline (0.1–64 µg ml^−1^), trimethoprim (0.5–32 µg ml^−1^), cefotaxime (0.5–32 µg ml^−1^), gentamicin (0.1–32 µg ml^−1^) and chloramphenicol (0.5–200 µg ml^−1^). The range of CORM-2 concentrations varied from 1 to 150 µg ml^−1^ (1.95–293 µg), at 5 µg ml^−1^ intervals. After the addition of antibiotics and CORMs, the plates were incubated for 24 h at 37 °C and 90 r.p.m. in a Sunrise^™^ microplate reader (Tecan, UK). MIC values were determined from the OD_600_ of each well. For the MBC determinations, 10 µl of each well that showed the MIC or higher was plated on LB agar and incubated for 72 h and the lowest concentration that prevented formation of colonies was considered as the minimum bactericidal concentration (MBC) value of each CORM.

### Checkerboard microdilution assay

Experiments were performed in 96-well plates as described previously [20,22]. Cultures were incubated at 37 °C with shaking for 24 h using a Sunrise^™^ microplate reader (Tecan, UK). Bacteria were grown in defined minimal medium with 5 g l^−1^ glucose as a carbon source to OD_600_ of 0.3. The concentrations of antibiotics tested were up to eightfold lower than the MIC and, where possible, twofold higher than the MIC. The MIC was considered the lowest concentration of the antibiotic alone or combined with CORMs that inhibited growth. The range of concentrations tested for CORMs and antibiotics was four dilutions lower than the MIC and two dilutions higher than the MIC. Fractional inhibitory concentration index (FICi) values were evaluated for each combination to determine drug interactions [22]. The MIC of each compound in combination, either CORM-2 or CORM-3, with an antibiotic, was defined as the lowest concentration of the individual agent that prevented visible bacterial growth in the presence of the other. The FICi was calculated as the sum of the individual FICs for each compound in the combination [22,23]:

FICi=FIC_CORM_ + FIC_antibiotic_

where FIC_CORM_=MIC of CORM in combination/MIC of CORM alone, and FIC_antibiotic_=MIC of antibiotic in combination/MIC of antibiotic alone.

Synergistic interactions were defined based on the FICi, where synergy was indicated by FICi≤0.5, partial synergy by values >0.5 and <1, additivity by FICi=1, indifference by FICi>1 and ≤4 and antagonism by FICi>4.

### Statistical analysis

All statistical analysis and data handling were performed using GraphPad Prism 5 (GraphPad Software). Differences between the conditions were tested by using a paired t-test.

## Results

### CORM-2 can act synergistically with antibiotics to increase antimicrobial activity

**Fig. 2.**
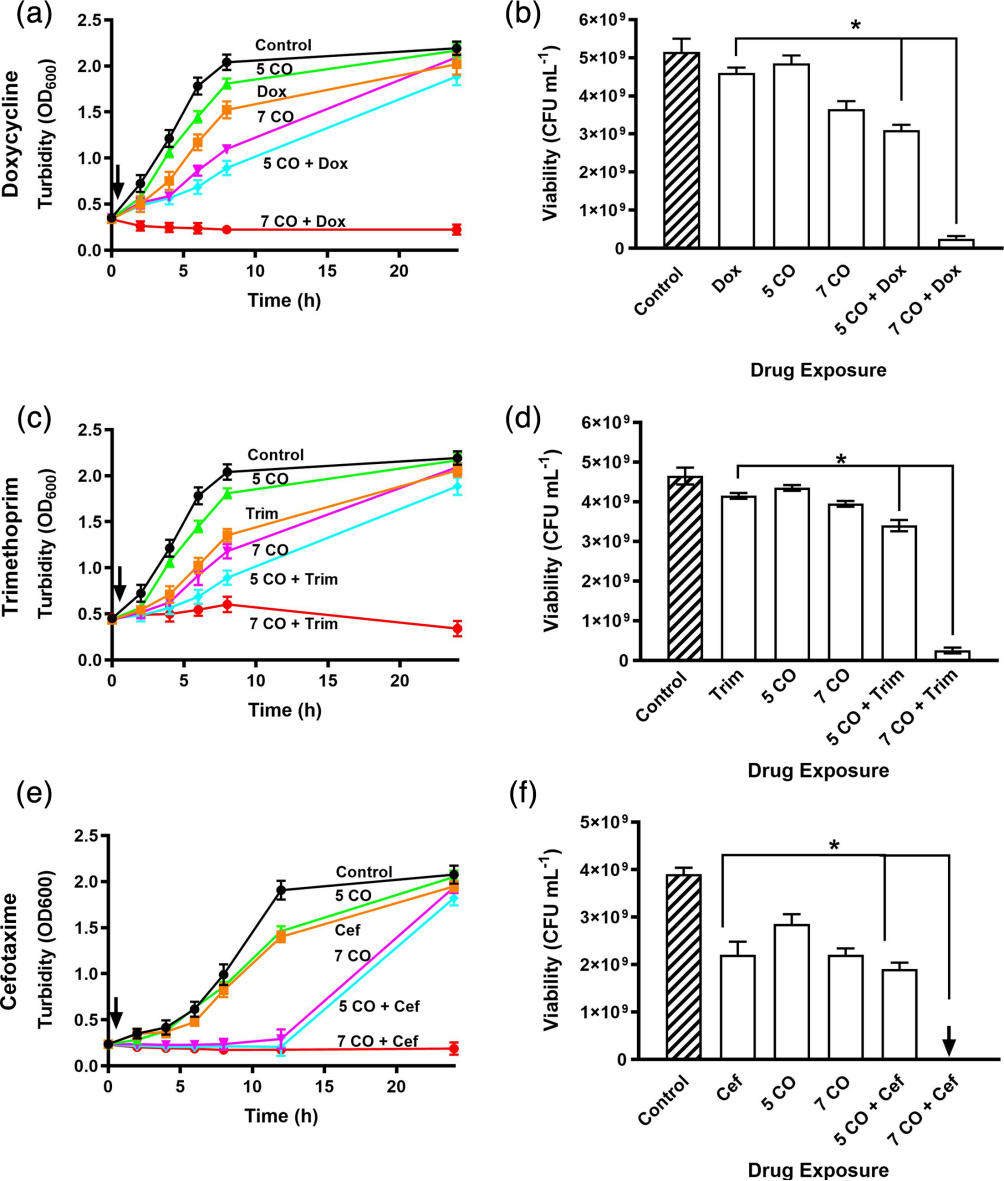
CORM-2 acts synergistically with antibiotics inhibiting growth and viability of *E. coli* MG1655. Cultures were treated with sublethal concentrations of CORM-2 (5 or 7 µM), either alone or in combination with (**a, b**) 1 µg ml^−1^ doxycycline, (**c, d**) 0.15 µg ml^−1^ trimethoprim or (**e, f**) 1 µg ml^−1^ cefotaxime. Growth inhibition was assessed by OD₆₀₀ measurements, and viability was determined after 24 h. Black lines and diagonal bar patterns represent control conditions; brown lines indicate treatment with antibiotics alone; green and magenta lines show treatment with CORM-2 alone; and cyan and red lines represent combined treatment with CORM-2 and antibiotics. Black arrows on growth curves represent the time of CORM-2 and/or antibiotic addition. Data points between 8 and 24 h are interpolated; cultures were sampled at 24 h for viability assessment, which confirmed the trends shown. Data are representative of three biological repeats and are expressed as means±sd. *P<0.05 (t-test).

The antimicrobial activity of CORMs against bacteria is well established [10,16, 17, 24], but their efficacy in combination with currently available antibiotics is less well understood. To investigate this, we selected two concentrations of CORM-2 (5 and 7 µM) that produced some growth inhibition without causing irreversible loss of viability after 24 h. In defined minimal medium, the untreated control exhibited a maximal specific growth rate (μmax) of ~0.23 h⁻¹ and the highest carrying capacities of the conditions tested (OD_600_ ~2.2, Fig. 2). CORM-2 alone reduced μmax to ~0.19 h⁻¹ (5 µM) and ~0.16 h⁻¹ (7 µM) but maintained carrying capacities similar to untreated controls, with neither concentration preventing recovery of viable cells. These doses were then combined with sublethal concentrations of antibiotics. Individually, doxycycline (~0.18 h⁻¹), trimethoprim (~0.16 h⁻¹) and cefotaxime (~0.27 h⁻¹) also altered growth rates but had little impact on the carrying capacities of the cultures. When combined with CORM-2, μmax decreased further over the first 8 h post-exposure: for doxycycline, growth was arrested with 7 µM and reduced to ~0.11 h⁻¹ with 5 µM; for trimethoprim, μmax fell to ~0.05 and ~0.10 h⁻¹ with 7 and 5 µM, respectively; and for cefotaxime, growth was completely arrested at both concentrations (Fig. 2). This reduction in growth is evident in the carrying capacities of the cultures. Treatment with any antibiotic and 5 µM CORM-2 resulted in a small decrease after 24 h, whereas combining antibiotic with 7 µM CORM-2 led to almost no detectable growth, corroborated by a significant reduction in viability. These findings indicate that CORM-2 substantially enhances antibiotic efficacy, prevents bacterial recovery and highlights its potential as an adjuvant to drugs with diverse mechanisms of action.

### CORM-3 can act synergistically with antibiotics to increase antimicrobial activity

**Fig. 3.**
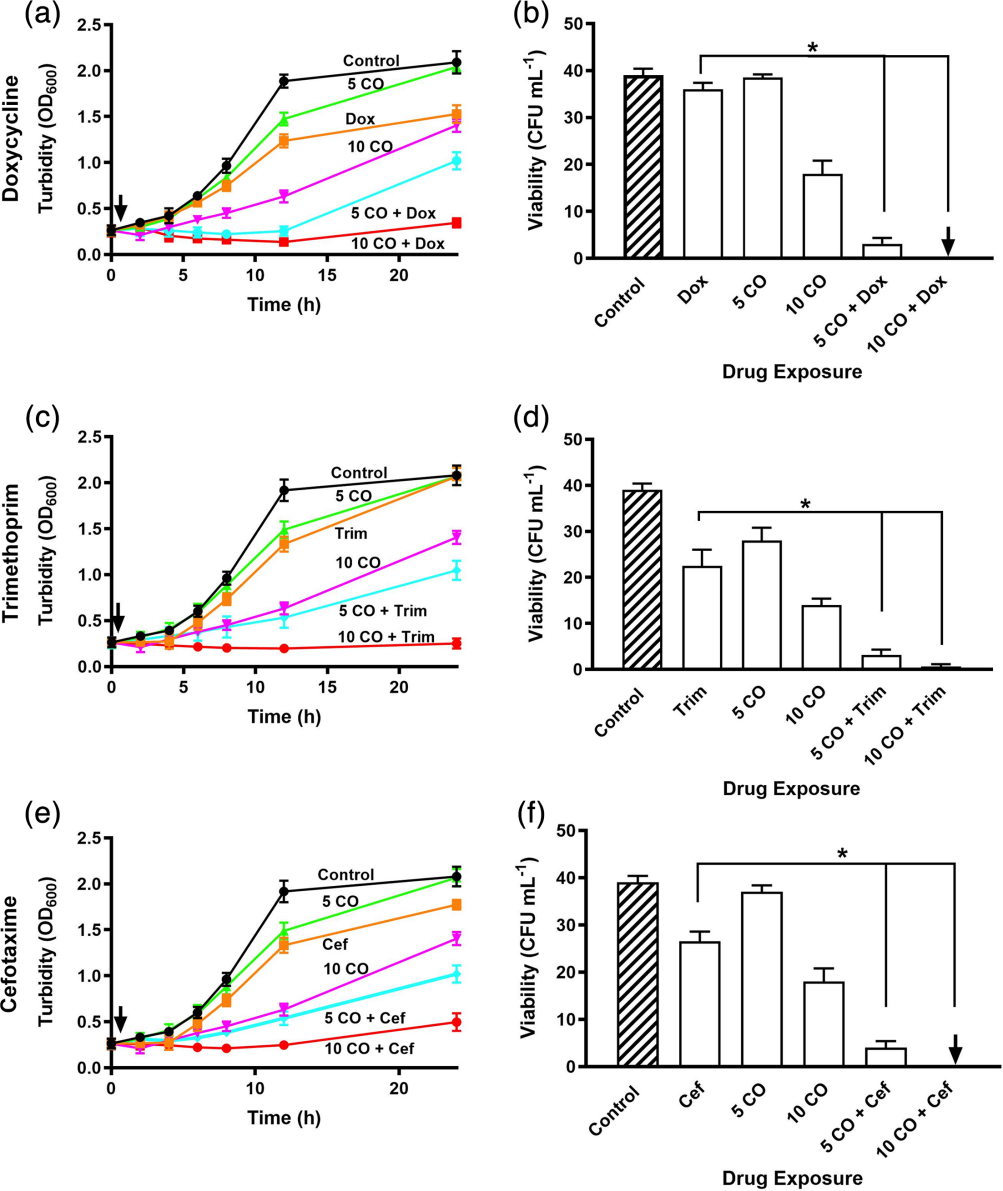
CORM-3 acts synergistically with antibiotics on growth and viability of *E. coli* MG1655. Cultures were treated with sublethal concentrations of CORM-3 (5 or 10 µM), either alone or in combination with (**a, b**) 1 µg ml^−1^ doxycycline, (**c, d**) 0.15 µg ml^−1^ trimethoprim or (**e, f**) 1 µg ml^−1^ cefotaxime. Growth inhibition was assessed by OD₆₀₀ measurements, and viability was determined after 24 h incubation. Black lines and diagonal bar patterns represent control conditions; orange lines indicate treatment with antibiotics alone; green and magenta lines show treatment with CORM-2 alone; and cyan and red lines represent combined treatment with CORM-2 and antibiotics. Black arrows represent time of CORM-3 and/or antibiotic addition. Data points between 12 and 24 h are interpolated; cultures were sampled at 24 h for viability assessment, which confirmed the trends shown. Data are representative of three biological repeats and are expressed as means±sd. *P<0.05 (t-test).

To determine whether the enhanced antimicrobial activity observed with CORM-2 was specific to its chemical properties, such as water insolubility, or a broader feature of CORMs, the water-soluble compound CORM-3 was tested for its ability to potentiate antibiotic efficacy. In defined minimal medium, active CORM-3 alone markedly reduced growth rates, with μmax falling to ~0.19 h⁻¹ at 5 µM and ~0.13 h⁻¹ at 10 µM. The carrying capacities of cultures exposed to 5 µM CORM-3 were similar to untreated controls, whereas exposure to 10 µM CORM-3 was markedly reduced (Fig. 3). When combined with sublethal doses of antibiotics, growth was almost completely suppressed within the first 12 h post-exposure: for doxycycline, μmax ranged from −0.04 to −0.10 h⁻¹; for trimethoprim, from 0.06 to −0.03 h⁻¹; and for cefotaxime, from 0.07 to −0.03 h⁻¹. These combinations also resulted in reduced carrying capacity and a significant reduction in bacterial viability, with little recovery after 24 h, particularly at 10 µM CORM-3 (Fig. 3). Collectively, these findings demonstrate that CORM-3, like CORM-2, can strongly potentiate the action of antibiotics with different mechanisms of action, highlighting the broader potential of CORMs as adjuvants.

### Inactivated CORMs and solvent controls confirm CO dependence of CORM antibacterial activity

**Fig. 4.**
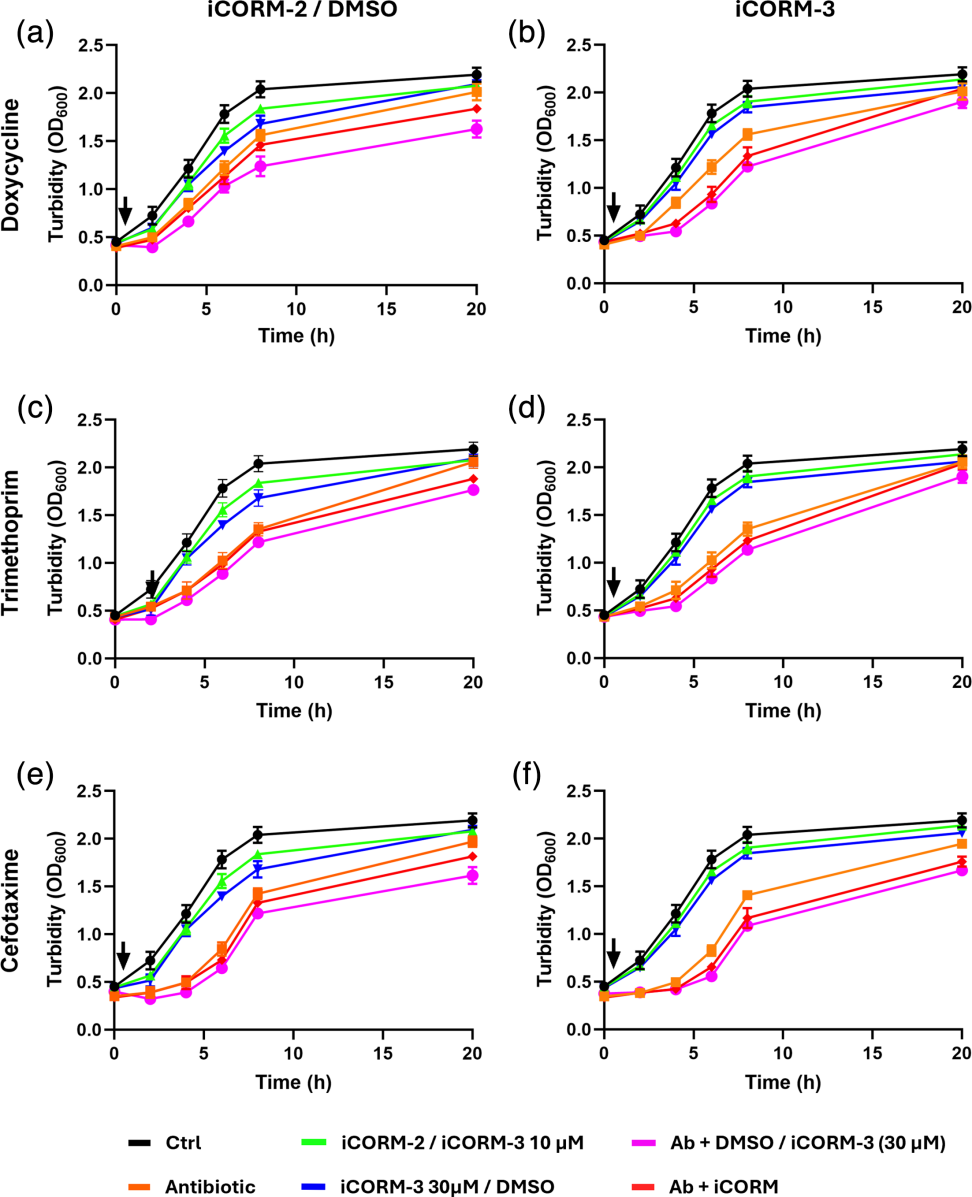
Inactivated CORMs and solvent controls show minimal *E. coli* growth inhibition and impact upon antibiotic activity. Growth of *E. coli* was assessed following treatment with antibiotics alone or in combination with either inactive CORM analogues (iCORM-2 or iCORM-3) or DMSO. Panels show the effects of (**a, b**) 1.0 µg ml⁻¹ doxycycline, (**c, d**) 0.15 µg ml⁻¹ trimethoprim and (**e, f**) 1.0 µg ml⁻¹ cefotaxime. In the left column (**a, c, e**), treatments included iCORM-2 and DMSO: black lines represent untreated controls, orange lines antibiotic alone, green lines iCORM-2 (10 µM), blue lines DMSO (equivalent to 10 µM iCORM-2), red lines antibiotic plus iCORM-2 and pink lines antibiotic plus DMSO. In the right column (**b, d, f**), treatments included iCORM-3 at 10 and 30 µM: black lines represent untreated controls, orange lines antibiotic alone, green lines iCORM-3 (10 µM), blue lines iCORM-3 (30 µM), red lines antibiotic plus iCORM-3 (10 µM) and pink lines antibiotic plus iCORM-3 (30 µM). Data represent the mean±sd of two biological replicates.

To assess whether the antibacterial effects of CORM-2 were attributable to its solvent (DMSO) or to the non-CO-releasing scaffold, we tested the effects of DMSO (at a concentration equivalent to that used to deliver 10 µM CORM-2) and 10 µM of the inactive analogue Ru(II)Cl₂(DMSO)₄ (iCORM-2), both alone and in combination with antibiotics. In defined minimal medium, the untreated control showed a maximal specific growth rate (μmax) of ~0.23 h⁻¹. Inactive controls showed similar growth rates (iCORM-2 ~0.24 h⁻¹; DMSO ~0.21–0.25 h⁻¹) and carrying capacities. Combinations with antibiotics produced only minor changes compared to antibiotics alone (e.g. doxycycline ~0.18–0.24 h⁻¹; trimethoprim ~0.16–0.18 h⁻¹; cefotaxime ~0.30–0.32 h⁻¹; Fig. 4a, c and e). These data confirm that neither DMSO nor the inactive scaffold contributed to growth inhibition or antibiotic potentiation. Higher µmax values observed in the presence of cefotaxime are likely a result of the significant inhibition of growth within the first 4 h post-exposure.

Similarly, we assessed whether the activity of CORM-3 was due to its ruthenium scaffold by testing the inactive form (iCORM-3) at 10 or 30 µM. Across conditions, μmax remained close to the untreated control (~0.23 h⁻¹), with negligible differences when combined with antibiotics (e.g. doxycycline ~0.19–0.20 h⁻¹; trimethoprim ~0.17–0.18 h⁻¹; cefotaxime ~0.29–0.33 h⁻¹) and carrying capacities remaining relatively stable (Fig. 4b, d and f). These findings demonstrate that the inhibitory and potentiating effects of both CORM-2 and CORM-3 depend on CO release rather than the solvent or metal scaffold.

### Sub-inhibitory CORM concentrations enhance antibiotic efficacy in E-test assays

The E-test (bioMérieux) is a widely used and reliable method for determining the MIC of antibiotics. To assess whether CORMs enhance antibiotic efficacy, we used this method to evaluate the effects of CORM-2 and CORM-3 in combination with various antibiotics against *E. coli*. In addition to broth assays, E-tests were employed to validate findings across both liquid and solid media, ensuring greater reproducibility and clinical relevance. Firstly, a range of CORM concentrations (10–100 µM) was tested in defined minimal medium agar to identify sub-inhibitory levels suitable for combination testing in the solid medium. Partial inhibitory concentrations were determined to be 30 µM for CORM-2 and 60 µM for CORM-3. These concentrations were then used in E-test assays to evaluate changes in antibiotic susceptibility.

**Fig. 5.**
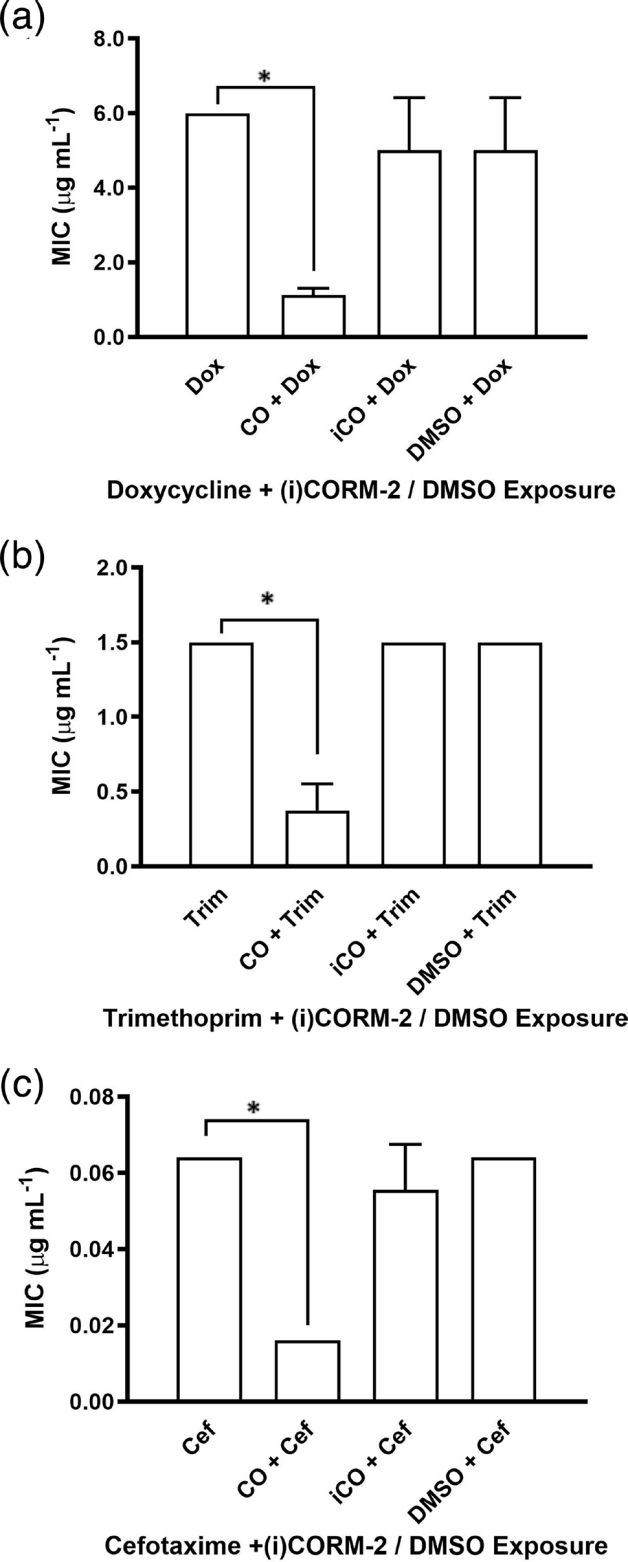
CORM-2 enhances the efficacy of selected antibiotics against *E. coli* in E-test MIC assays. E-tests were performed on *E. coli* grown on defined minimal medium agar to assess the impact of 30 µM CORM-2 on susceptibility to (**a**) doxycycline, (**b**) trimethoprim and (**c**) cefotaxime. The data are representative of three biological repeats and are expressed as mean±sd. *P<0.05 (t-test). Where error bars are not shown, this indicates that the MIC result was the same in all repeats.

**Fig. 6.**
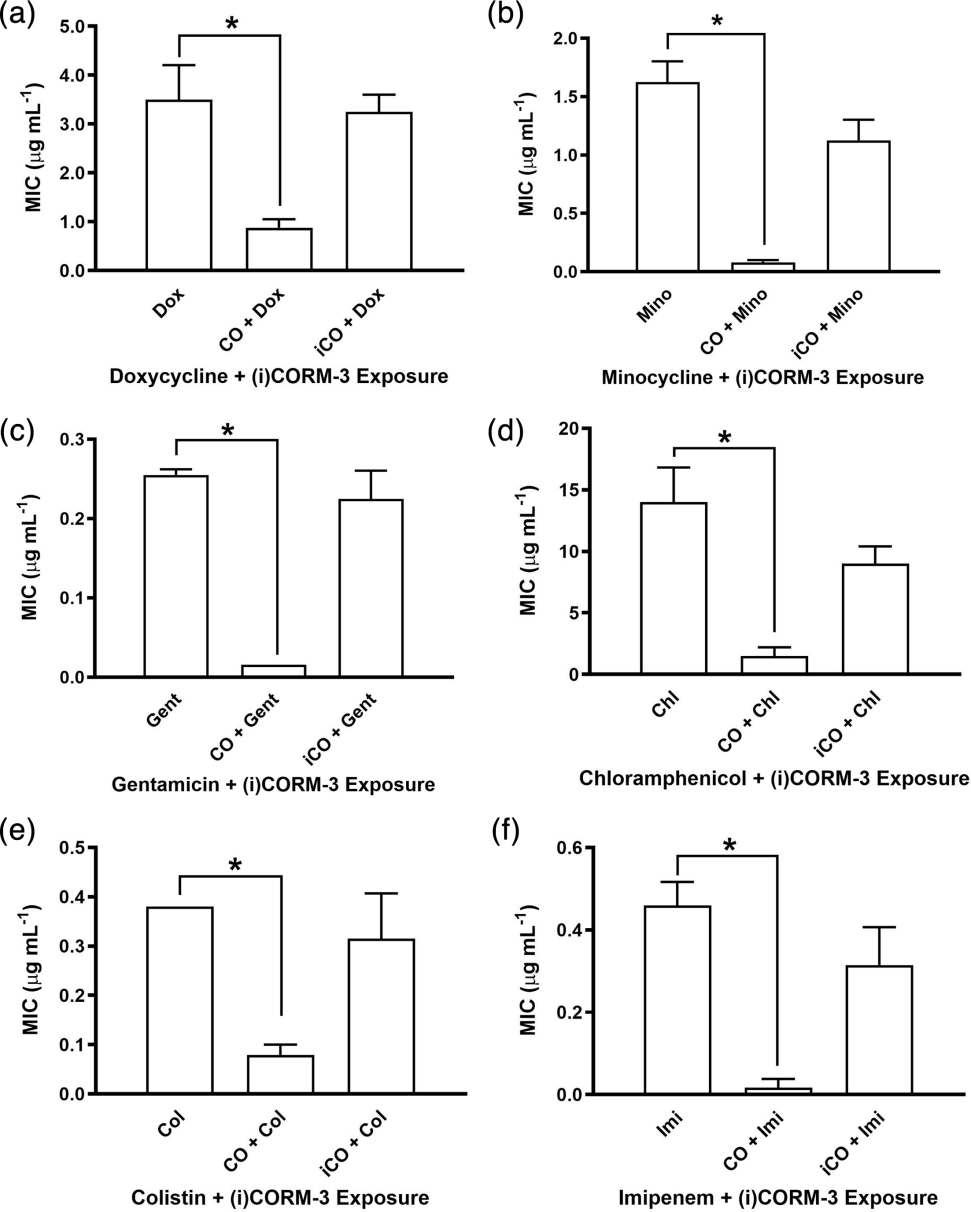
CORM-3 potentiates the activity of antibiotics from diverse classes in E-test MIC assays. To evaluate the broader applicability of CORMs, E-tests were conducted against *E. coli* using 60 µM CORM-3 in combination with six antibiotics representing multiple classes – (**a**) doxycycline, (**b**) minocycline, (**c**) gentamicin, (**d**) chloramphenicol, (**e**) colistin and (**f**) imipenem. The data are representative of three biological repeats and are expressed as mean±sd. *P<0.05 (t-test). Where error bars are not shown, this indicates that the MIC result was indistinguishable in all repeats.

The presence of 30 µM CORM-2 resulted in a twofold to sixfold reduction in the MIC values of doxycycline, trimethoprim and cefotaxime (Fig. 5). This effect was not observed with either DMSO or the inactive analogue iCORM-2, highlighting the requirement for CO release in mediating the antimicrobial enhancement. To determine whether this effect was limited to the initial three antibiotics or extended more broadly, we tested 60 µM CORM-3 against six antibiotics from an expanded range of classes, including the carbapenems, polymyxins and aminoglycosides. CORM-3 reduced the MICs of all six antibiotics by 2- to 14-fold (Fig. 6), whereas iCORM-3 had no impact, further supporting the role of CO release in potentiating antibiotic activity. These findings demonstrate that both CORM-2 and CORM-3 can enhance the activity of antibiotics with diverse mechanisms of action, likely through their CO-releasing properties.

### CORMs enhance antibiotic efficacy through minimum bactericidal concentration reduction

**Fig. 7.**
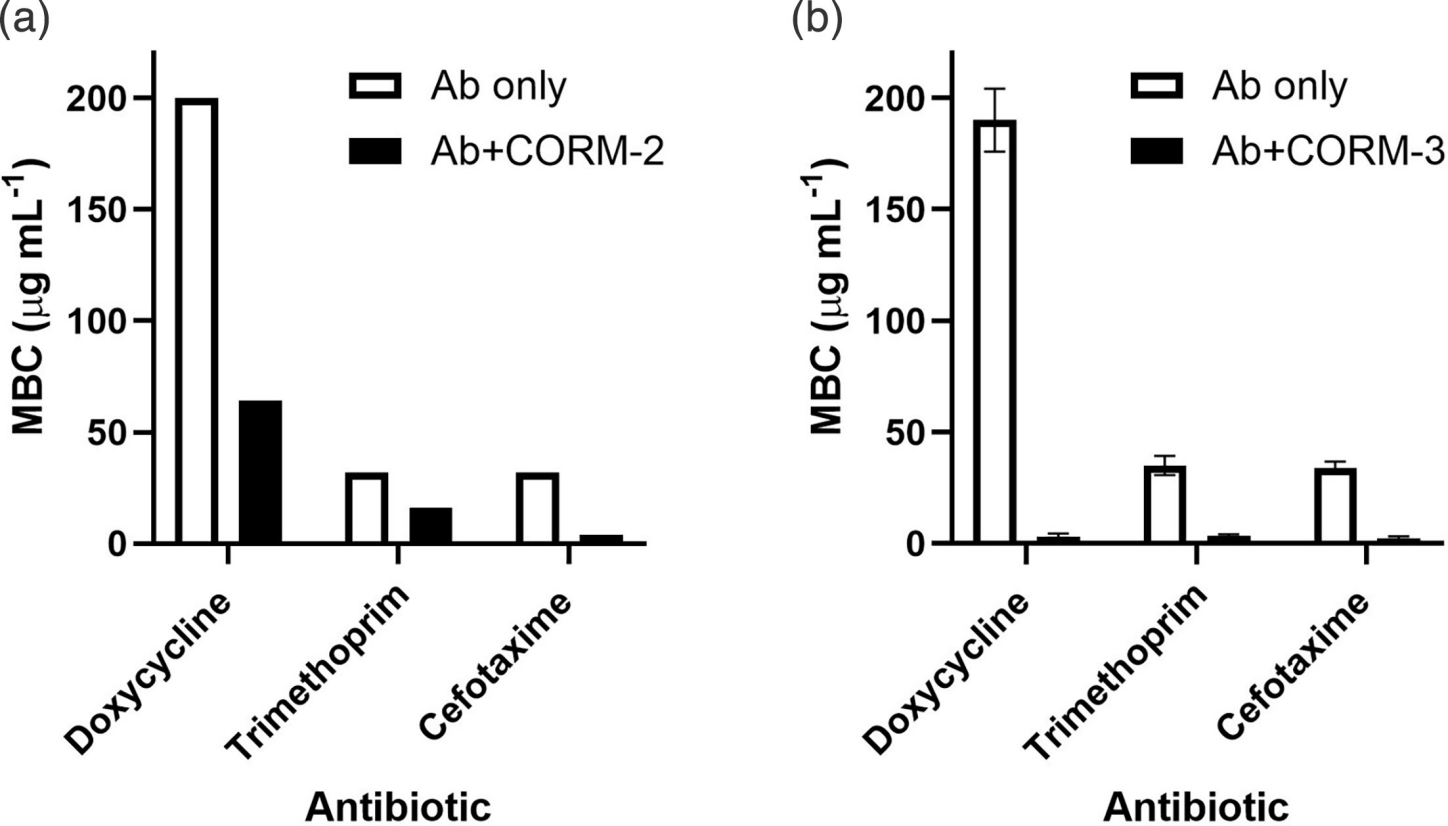
Sublethal concentrations of CORM-2 and CORM-3 reduce minimum bactericidal concentration values of antibiotics against *E. coli*. MBC assays were performed in the presence or absence of (**a**) CORM-2 (5 µg ml^−1^; 9.76 µM) or (**b**) CORM-3 (15 µg ml^−1^; 50.91 µM) to quantify their impact on antibiotic susceptibility. The data are representative of three biological repeats and are expressed as mean±sd. Where error bars are not shown, this indicates that MBC results were the same in all repeats.

To further quantify the impact of CORMs on antibiotic susceptibility, we performed minimum bactericidal concentration assays in the presence and absence of CORM-2 and CORM-3 at their MIC values (5 µg ml⁻¹/9.76 µM and 15 µg ml⁻¹/50.91 µM, respectively). This method enabled precise measurement of changes in MBC values and confirmed the potentiating effects observed in earlier assays (Figs2, 3, 5  6). In the presence of CORM-2, MBC values were reduced twofold to eightfold (Fig. 7a). Similarly, addition of CORM-3 led to reductions in antibiotic MBCs ranging from 10- to 63-fold (Fig. 7b). Notably, the concentration of doxycycline required for bactericidal activity decreased 63-fold (Fig. 7b). These results demonstrate that sublethal concentrations of CORMs can markedly enhance the efficacy of antibiotics with diverse mechanisms of action, significantly lowering both MIC and MBC values against *E. coli*.

### Synergistic effects of CORMs with selected antibiotics revealed by checkerboard assays

To further investigate the nature of the interaction between CORMs and antibiotics, we next employed checkerboard microdilution assays. This method allows for systematic evaluation of drug interactions by calculating the FICi, which quantifies the combined effect of two agents relative to their individual MICs. A FICi value ≤0.5 indicates synergy, 0.5–4 indicates an additive or indifferent effect and >4 suggests antagonism.

Using this approach, we found that CORM-2 exhibited synergistic interactions with several protein synthesis inhibitors, including trimethoprim (FICi=0.41), novobiocin (0.35), doxycycline (0.37) and spectinomycin (0.50). In contrast, no interaction was observed with cell wall synthesis inhibitors such as ampicillin and cefotaxime (FICi=1.25 for both; Table 1). Similarly, CORM-3 showed synergistic effects with doxycycline, trimethoprim, minocycline and cefotaxime, with FICi values ranging from 0.31 to 0.45 (Table 1). These results support the potential of CORMs to selectively enhance the activity of certain antibiotics, particularly those targeting protein synthesis.

**Table 1.** Synergistic interactions between CORMs and antibiotics determined by checkerboard microdilution assays MIC is expressed in μg ml^−1^.

CORM	Antibiotic	FIC _antibiotic_	FIC _CORM_	Σ FIC	Combination interaction
CORM-2	Trimethoprim	0.16	0.25	0.41	Synergy
	Novobiocin	0.10	0.25	0.35	Synergy
	Doxycycline	0.12	0.25	0.37	Synergy
	Spectinomycin	0.25	0.25	0.50	Synergy
	Ampicillin	1.00	0.25	1.25	No interaction
	Cefotaxime	1.00	0.25	1.25	No interaction
CORM-3	Doxycycline	0.13	0.25	0.38	Synergy
	Trimethoprim	0.20	0.25	0.45	Synergy
	Minocycline	0.13	0.25	0.38	Synergy
	Cefotaxime	0.06	0.25	0.31	Synergy

Values are means of three independent biological repeats.

## Discussion

The emergence of multidrug-resistant pathogenic bacteria has posed a major, growing public health risk, with observers claiming that we are approaching the ‘post-antibiotic era’ [25]. Prior to the work described in this study, the antibacterial activity of CORM-2 and CORM-3 was demonstrated for several Gram-negative and Gram-positive bacterial species [10,16, 17, 26, 27]. However, there is no evidence that CORMs alone could act as effective antibacterial agents in therapy. Therefore, it was important to investigate the potential effects of CORMs on antibiotic efficacy against bacteria. Our findings with a model bacterium show that a combination of sublethal concentrations of either CORM-2 or CORM-3 significantly potentiates the action of multiple antibiotics with different modes of action. In this study, we selected antibiotics from multiple classes with distinct mechanisms of action, including protein and cell wall synthesis inhibitors. These agents are commonly used in the treatment of *E. coli* infections and provide a representative spectrum for assessing whether CORMs exert broad synergistic effects across different antibiotic targets. The finding that inactive forms (iCORM-2 or iCORM-3) did not display these effects suggests that CO release is crucial to antimicrobial activity. The minimal increases in antibacterial activity observed in Fig. 4 are likely the result of residual active CORM found in the preparation [27]. However, recent findings have questioned the role of CO in the biological effects of the Ru-containing CORMs and instead implicated the Ru(II) as a dominant factor in biotoxicity [28,29]. Bacteria were protected from the toxic effects of these CORMs by inclusion in the media of amino acids and thiols. In the present work, wholly defined media with no complex components were used, suggesting that full CORM toxicity should be expressed. These papers also showed that toxicity to *E. coli* was highly correlated with cellular accumulation of Ru, which becomes bound to DNA [30]. Therefore, we speculate that the presence of antibiotics may increase the permeability of the cell envelope to CORM-3 or CORM-2 but not to the control molecules tested, namely RuCl_2_(DMSO) and CO-depleted CORM-3. Further studies of the intracellular transport of CORMs and their ‘inactive’ counterparts are required to test this hypothesis.

Whatever the mechanisms of co-toxicity, our results showed that partial inhibitory concentrations of CORM-3 significantly potentiate the activity of several antibiotics with different modes of action in killing bacterial cells. Similar findings have been reported by Tavares *et al.*, who found that CORM-2 (200 mg l^−1^) significantly decreased the viability of *Helicobacter pylori* when combined with partial inhibitory concentrations of metronidazole [31]. Another investigation revealed that a novel manganese photoactivated CORM (at 200 µM) had a slight potentiating effect on the activity of doxycycline by reducing the growth of *E. coli* EC958 [24].

The results herein revealed that iCORMs had little effect on bacterial growth or antibiotic potentiation. This result is in agreement with a study that found ruthenium chloride does not alter bacterial growth at concentrations ranging from 1 to 10 µM [17]. Other researchers, using a microdilution susceptibility test, found that exposure up to 400 mg l^−1^ of Ru(II)Cl_2_(DMSO)_4_ had no effect on the growth and viability of *H. pylori* and did not affect the potentiation of metronidazole [31]. Furthermore, the study by Murray *et al.* [32] showed that neither the inactive form Ru(II)Cl_2_(DMSO) nor DMSO had an effect on the planktonic growth of *P. aeruginosa* [32]. Further, Bang and colleagues found that 500 µM Ru(II)Cl_2_(DMSO) had no effect on growth and viability of multidrug-resistant uropathogenic *E. coli* [33].

Control experiments confirmed that neither DMSO nor inactive CORM analogues (iCORM-2 and iCORM-3) influenced antibiotic activity against *E. coli*, supporting the conclusion that CO release is critical for the observed potentiation. This finding aligns with previous reports that synergy in combination therapies depends on the active component rather than inactive scaffolds [34,36], underscoring the mechanistic importance of CO release in CORM-mediated antibacterial effects.

The use of sublethal concentrations of CORMs, as in our study, may offer an advantage to high dose single therapies by limiting systemic exposure and reducing potential *in vivo* toxicity [14,15, 17, 29]. Although short-term administration of ruthenium-based CORMs in animal models has generally been well tolerated, systemic ruthenium exposure can lead to off-target effects, including DNA binding and cytotoxicity [29,30]. Carbon monoxide itself has a therapeutic precedent, having been explored for anti-inflammatory and cytoprotective effects under controlled delivery, suggesting that regulated CO release may be clinically acceptable [14,15]. Moreover, ruthenium complexes are already employed in oncology as chemotherapeutic agents, indicating that regulatory pathways for Ru-based compounds exist, albeit with stringent safety requirements [37]. Future development of CORMs as antibiotic adjuvants will require careful optimization of dosing strategies to minimize toxicity, alongside comprehensive pharmacokinetic and toxicological evaluations to meet regulatory standards for clinical use. Our data show that sub-inhibitory concentrations of CORMs markedly lower MICs and MBCs across multiple antibiotic classes (Fig. 7), consistent with a broad adjuvant effect that could help restore the efficacy of existing agents against *E. coli*. Mechanistically, CORMs act via multitarget perturbations (e.g. respiration, ion transport, sulphur biochemistry and DNA interactions), which may reduce the probability of single-target resistance [11,16, 27, 30]. The principal limitations relate to toxicity and delivery: while short-term administration of Ru-based CORMs has been generally tolerated in animal models, ruthenium accumulation and DNA binding can contribute to off-target effects [14,15, 17, 29, 30]. Controlled CO release has recognized anti-inflammatory and cytoprotective profiles that may be acceptable under regulated dosing [14,15], but clinical translation will require infection-specific dosing, pharmacokinetic and toxicological evaluations to balance antimicrobial benefit against systemic exposure [14,15, 17, 29, 30].

Although our experiments were mostly performed in planktonic *E. coli* cultures, several studies indicate that CORMs can impair biofilm formation and viability, particularly in clinically relevant pathogens. CORM2 attenuates *P. aeruginosa* biofilms and reduces associated virulence in an *in vivo* bacteraemia model [32], while EBORCORM1 exhibits antimicrobial activity shaped by population variation in *P. aeruginosa* [12]. In uropathogenic *E. coli*, CORM2 inhibits biofilm growth following host cell colonization [33]. In addition, photoactivable manganese-based CORMs are active against ESKAPE-derived biofilms [13]. These data support a rationale for testing CORM–antibiotic combinations in biofilm-mimicking conditions, particularly for complicated urinary tract infections.

While classical, stable target-specific resistance (as seen with single-target antibiotics) may be less likely due to the multimodal actions of CORMs, tolerance and decreased susceptibility are plausible through adaptive responses (e.g. thiol/amine scavenging, oxidative stress responses), altered permeability/efflux or modulation of ruthenium accumulation [11,27, 29, 30]. Indeed, intraspecies variation in *P. aeruginosa* influences EBORCORM1 activity [12], suggesting a pathway for selection under prolonged exposure. To address this, future work should include serial passage evolution with CORMs in the presence and absence of antibiotics, assays of efflux pump activity, quantification of intracellular ruthenium and carbon monoxide and whole-genome transcriptomic analyses to identify adaptive trajectories [11,12, 27, 29, 30].

Enhancing antibiotic activity through combination with other antimicrobial compounds is a recognized strategy to tackle resistance [38]. For example, antimicrobial peptides such as novicidin have shown synergy with rifampicin and cephalosporins against multidrug-resistant *Enterobacteriaceae* [39]. Importantly, CORM-based conjugates with azole antibiotics, such as [Mn(CO)₃(2,2′-bipyridyl)(azole)]^+^ and [Re(CO)₃(Bpy)(Ctz)]^+^, exhibit potent antibacterial activity far exceeding their individual components, with activity against both Gram-positive and Gram-negative bacteria [40].

Future investigations should explore CORM synergy with critical last-line antibiotics in an infection-specific manner, for example, assessing the impact of co-delivery of carbapenems with CORMs under conditions that mimic complicated urinary tract infections, where carbapenems are frequently employed for multidrug-resistant *Enterobacteriaceae*.

In conclusion, CORMs exhibit significant antibacterial activities, particularly in combination with various antibiotics with different modes of action. Therefore, CORMs may be suitable antibacterial compounds to be used as adjuvants to existing antibiotics to combat multidrug-resistant bacteria. Further studies are needed to understand the modes of action for CORMs in isolation and in combination with better established antimicrobial compounds.

## Abbreviations

CORMs: carbon monoxide-releasing molecules
FICi: fractional inhibitory concentration index
LB: Luria–Bertani
MBC: minimum bactericidal concentration
